# Fenfluramine diminishes NMDA receptor-mediated seizures via its mixed activity at serotonin 5HT2A and type 1 sigma receptors

**DOI:** 10.18632/oncotarget.25169

**Published:** 2018-05-04

**Authors:** María Rodríguez-Muñoz, Pilar Sánchez-Blázquez, Javier Garzón

**Affiliations:** ^1^ Neuropharmacology, Department of Translational Neurosciences, Cajal Institute, CSIC, Madrid E-28002, Spain

**Keywords:** fenfluramine, seizures, glutamate N-methyl-D-aspartate receptor, type 1 sigma receptor, HINT1 protein

## Abstract

Fenfluramine exhibits antiepileptic properties and thus diminishes epileptiform discharges in experimental animal models of Dravet syndrome. Fenfluramine is metabolized into norfenfluramine *in vivo*, which shows greater affinity and agonist activity at serotonin 5HT2 receptors (5HT2R) than fenfluramine. In this study, we found that fenfluramine and norfenfluramine disrupted the regulatory association of the sigma 1 receptor (σ1R) with NR1 subunits of glutamate *N*-methyl-D-aspartate receptors (NMDAR), an effect that was also produced by σ1R antagonists such as S1RA and prevented by σ1R agonists such as PPCC. The antagonists removed σ1R bound to NMDAR NR1 subunits enabling calcium-regulated calmodulin (CaM) to bind to those subunits. As a result, CaM may inhibit calcium permeation through NMDARs. The serotoninergic activity of fenfluramine at 5HT2AR, and likely also at 5HT2CR, collaborated with its activity at σ1Rs to prevent the convulsive syndrome promoted by NMDAR overactivation. Notably, fenfluramine enhanced the inhibitory coupling of G protein-coupled receptors such as 5HT1AR and cannabinoid type 1 receptor with NMDARs, thus allowing the more effective restrain of NMDAR activity. Thus, fenfluramine circumvents the negative side effects of direct NMDAR antagonists and may improve the quality of life of subjects affected by such proconvulsant dysfunctions.

## INTRODUCTION

Altered central inhibitory (*e.g*., γ-aminobutyric acid, or GABA) and excitatory (*e.g*., glutamate) neurotransmission is well established to play a pivotal role in the etiology of epilepsy, with excess glutamatergic transmission and the ensuing overactivation of glutamate receptors being particularly relevant to its clinical manifestations [1–3]. Thus, during prolonged seizures, the activity of GABA-A receptors decreases, causing increases in the activity of glutamatergic *N*-methyl-D-aspartate (NMDA) receptors, which often results in refractory status epilepticus [4]. Calcium ions pass through the NMDA ionotropic receptor into neural cells, and NMDA receptor activity is regulated by a series of neurotransmitters and cytosolic proteins to establish synaptic tonus through the undulating transitions between long-term potentiation and long-term depression.

Unfortunately, in epilepsy, NMDAR stimulation escapes from these controls responsible for maintaining excitatory activity within physiological limits, and its deregulation plays a key role in the generation of seizures. NMDAR deregulation may be a consequence of upstream dysfunctions such as those that affect diverse epileptogenic manifestations, including devastating forms of childhood epilepsy that begin with prolonged seizures in the first year of life such as West [5], Dravet [6, 7] and Lennox-Gastaut syndromes [8, 9]. Competitive and noncompetitive NMDAR channel blockers exhibit potent anticonvulsant activity in animal models [10–12]. Accordingly, ketamine and memantine appear to be effective for the control of multidrug-resistant epilepsy in children and adults [4]. Nevertheless, because of side effects, the treatment of epilepsy with chronic selective NMDAR antagonists has mostly disappointed in clinical trials [13].

Fenfluramine (3-trifluoromethyl-N-ethylamphetamine), originally recognized as an appetite suppressant in the mid-1960s [14], was used as an add-on drug and demonstrated to be a potent antiepileptic drug in Dravet syndrome as 70% of the children were seizure-free for more than 1 year [15, 16]. Phase 3 clinical trials with ZX008 are currently ongoing in the USA and Europe (Zogenix: low-dose fenfluramine - orphan drug designation granted for ZX008 in Dravet syndrome by the FDA). Fenfluramine is a racemic mixture of two enantiomers: dexfenfluramine and levofenfluramine, which *in vivo* are metabolized to d(+)-(6)-norfenfluramine and l(-)-(1)-norfenfluramine, respectively [17]. The affinity and agonist activity exhibited by d-norfenfluramine at serotonin 5HT2A/B/C receptors are better than those of the l-metabolite [18, 19]. In the brain, particular members of the 5HT2 receptor family such as 5HT2A and 5HT2C are their likely targets [18]. Because, with a few exceptions, the anticonvulsant activity of the most selective agonists and antagonists at 5-HT receptor subtypes remains controversial [20], the exact underlying mechanism of the anticonvulsant activity of fenfluramine is still not known, and an interaction at other receptors cannot be ruled out at this time.

The activity of glutamate NMDARs falls under the negative influence of some G protein-coupled receptors (GPCRs) including the cannabinoid type 1 receptor (CB1R) [21], the acetylcholine type 1 muscarinic receptor [22], the serotonin 5HT1A receptor [23], the adrenergic α1 and α2 receptors [24], the dopamine D3 and D4 receptors [25, 26], and the group III mGluR7 receptors [27]. However, there are GPCRs that enhance NMDAR calcium flux by means of non-receptor tyrosine kinases such as Src and Fyn [28] and through serine and threonine kinases such as PKC and PKA [29, 30]. These GPCRs include the mu opioid receptor (MOR) [31], the dopamine D1 receptor [32, 25], the group I (mGluR1/5) and group II (mGluR2/3) metabotropic glutamate receptors [33, 34], and the serotonin 5HT2A/C receptors [35].

In this context, the complex formed by the histidine triad nucleotide-binding protein 1 (HINT1) and the sigma receptor type 1 (σ1R) supports the physical coupling and uncoupling between GPCRs such as CB1R or MOR with NMDARs [36, 37]. Thus, the HINT1-σ1R protein complex connects and disconnects the activity of GPCRs with that of NMDARs. Although the role of σ1Rs in the pathophysiology of epilepsy has not been fully established, some σ1R ligands such as dextrorphan and carbetapentane ameliorate status epilepticus induced by kainic acid [38, 39], and racemic (±)-pentazocine antagonizes electrical tonic convulsions in mice [40]. Likewise, recent data on the involvement of σ1Rs in rare CNS diseases highlights the potential of the sigma ligand ANAVEX 2-73 to treat epilepsy [41]. Additional studies with highly selective σ1R ligands would definitely shed some light on their therapeutic potential as anticonvulsive agents.

Against this background, we addressed whether fenfluramine displays activity at σ1Rs to affect their regulatory interaction with NMDAR NR1 subunits in an *in vitro* model [37, 36]. In *ex vivo* assays performed in mouse brain synaptosomes, we also determined the influence of *in vivo* administration of fenfluramine on the inhibitory association that certain GPCRs, such as 5HT1A and CB1, establish with NMDARs via NR1 subunits. Our data suggest that fenfluramine direct activity at 5HT2Rs and σ1Rs, and indirect at GPCRs such as CB1R and 5HT1AR, restrains NMDAR activity effectively reducing the severity of the convulsing syndrome.

## RESULTS

### Activity of fenfluramine on σ1Rs

The function of glutamate NMDARs can be modulated via the σ1R on the plasma membrane [42]. In cells, the σ1R physically interacts with the NMDAR NR1 but not with the NR2A subunit, and the NR1 subunit only has a single σ1R binding site [43], which is located in its cytosolic C terminal regulatory region [36]. The interaction of σ1Rs with NMDAR NR1 subunits is calcium-dependent [37, 44], and it has been mapped onto the same cytosolic region that binds calcium-activated calmodulin (CaM) to reduce the open probability of the calcium channel [45].

**Figure 1:**
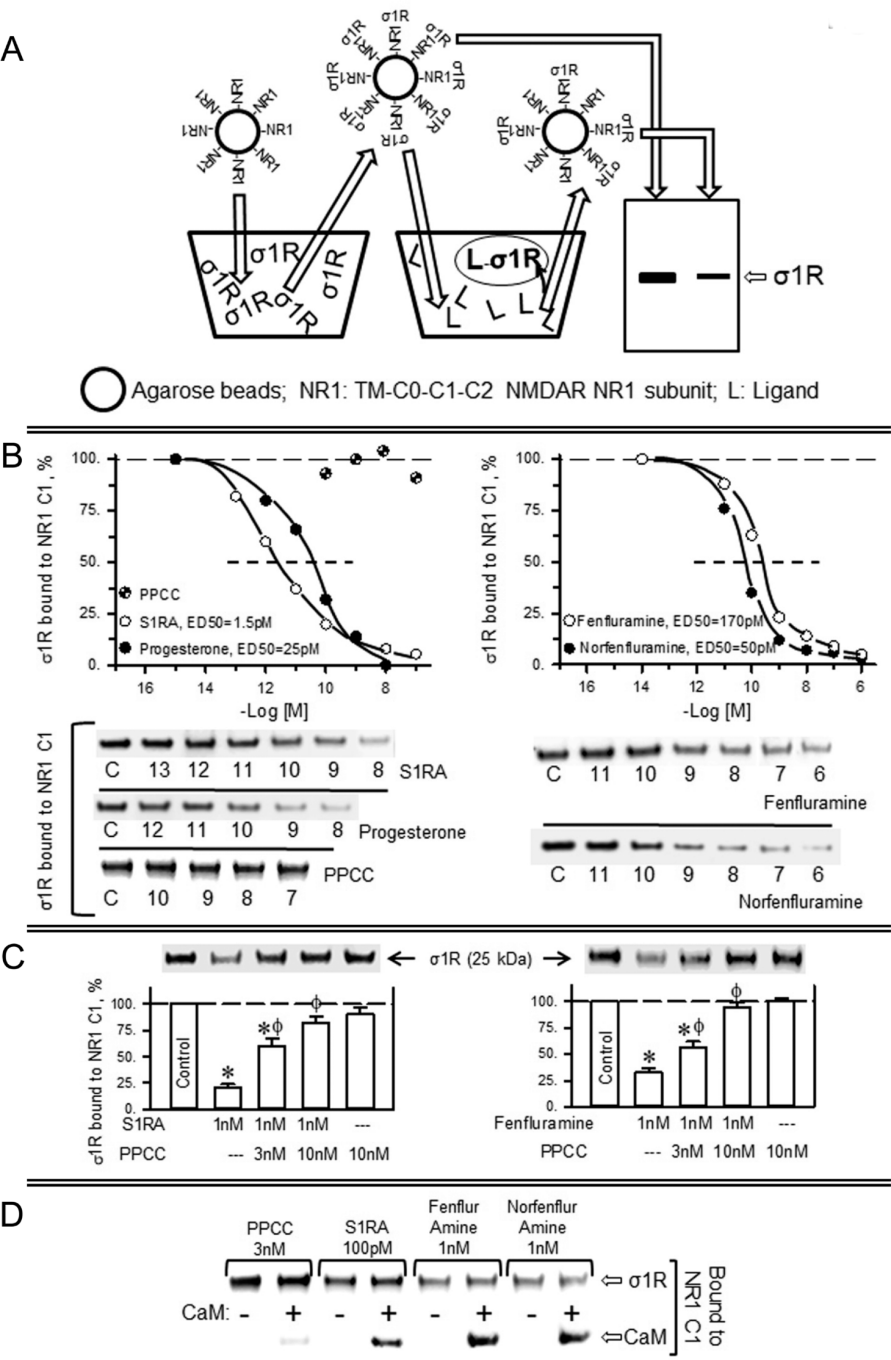
Fenfluramine and norfenfluramine disrupt the association of σ1Rs with NR1 subunits of NMDA receptors (**A**) *In vitro* assay to determine ligand activity at σ1Rs. NHS-activated Sepharose beads covalently coupled to a sequence of NR1 subunits containing the transmembrane region plus C0–C1–C2 cytosolic segments were incubated with excess σ1Rs (1:3). The unbound σ1Rs were washed out, and the NR1-coupled σ1Rs were exposed to serial concentrations of the ligands under study. The σ1Rs that remained attached to the NR1 subunits were then evaluated by SDS-PAGE and immunoblotting. (**B**) Diminishing effect of ligands on the association of σ1Rs with NR1 subunits. The assays were performed in the presence of 50 mM Tris–HCl (pH 7.5), 0.2% CHAPS and 2.5 mM calcium. Representative blots are shown. The ED_50_ values were computed using the software SigmaPlot V.13. (**C**) The σ1R agonist PPCC diminishes the capacity of S1RA and fenfluramine to disrupt σ1R–NR1 association. (**D**) S1RA, fenfluramine and norfenfluramine, but not PPCC, decreased the association of σ1Rs with CaM binding site on NR1 subunits. Agarose-NR1 carrying the associated σ1Rs was incubated with 2.5 mM CaCl_2_, in the absence or presence of 100 nM CaM. The assays were performed twice, and each point was duplicated. ^*^Significant difference with respect to the control group, ϕ with respect to the group receiving only S1RA or fenfluramine; ANOVA, Dunnett multiple comparisons vs control group, *p* < 0.05.

Thus, in an *in vitro* assay, we addressed the capacity of σ1R ligands to alter the interaction of recombinant σ1Rs with the regulatory cytosolic C0-C1-C2 region of NMDAR NR1 subunits. In this paradigm, the σ1R ligands display concentration-dependent activity on the quality of the σ1R-NR1 association; thus, antagonists disrupt the σ1R-NR1 association, and agonists prevent this effect of the antagonists [36, 37]. The last transmembrane region of the NR1 subunit spaced the C0-C1-C2 region from its covalent attachment to agarose particles (see Methods). Thus, agarose-NR1 was incubated with σ1Rs, and after the removal of those not bound to NR1 subunits, the pre-formed agarose-NR1-σ1R complexes were then exposed to the effects of potential σ1R ligands. The σ1Rs that remained bound to the agarose-NR1 subunits were subsequently evaluated (Figure 1A).

The endogenous neurosteroid progesterone, a putative σ1R antagonist, exhibited an apparent ED_50_ of 25 pM for diminishing the presence of NR1-σ1R complexes. The most selective σ1R antagonist, S1RA [46], displayed an ED_50_ of approximately 1.5 pM, whereas the agonist PPCC had no effect in this context. Notably, (+)-fenfluramine and its metabolite (+)-norfenfluramine disrupted NR1-σ1R associations with an ED_50_ of 170 pM and 50 pM, respectively. Both serotoninergic substances displayed a dose-dependent capacity to diminish the NR1-σ1R association and thus behaved as σ1R antagonists (Figure 1B). The effects of 1 nM S1RA and of 1 nM fenfluramine on impairing the NR1-σ1R association were diminished by 3 nM and 10 nM of the σ1R agonist PPCC (Figure 1C). The binding of the σ1R to the NR1 subunit blocks the access to the negative regulator of NMDAR function, CaM [36]. S1RA, fenfluramine and norfenfluramine, but not the agonist PPCC, reduced σ1R binding to NR1 subunits and consequently increased the calcium-dependent association of CaM with these subunits (Figure 1D).

**Figure 2:**
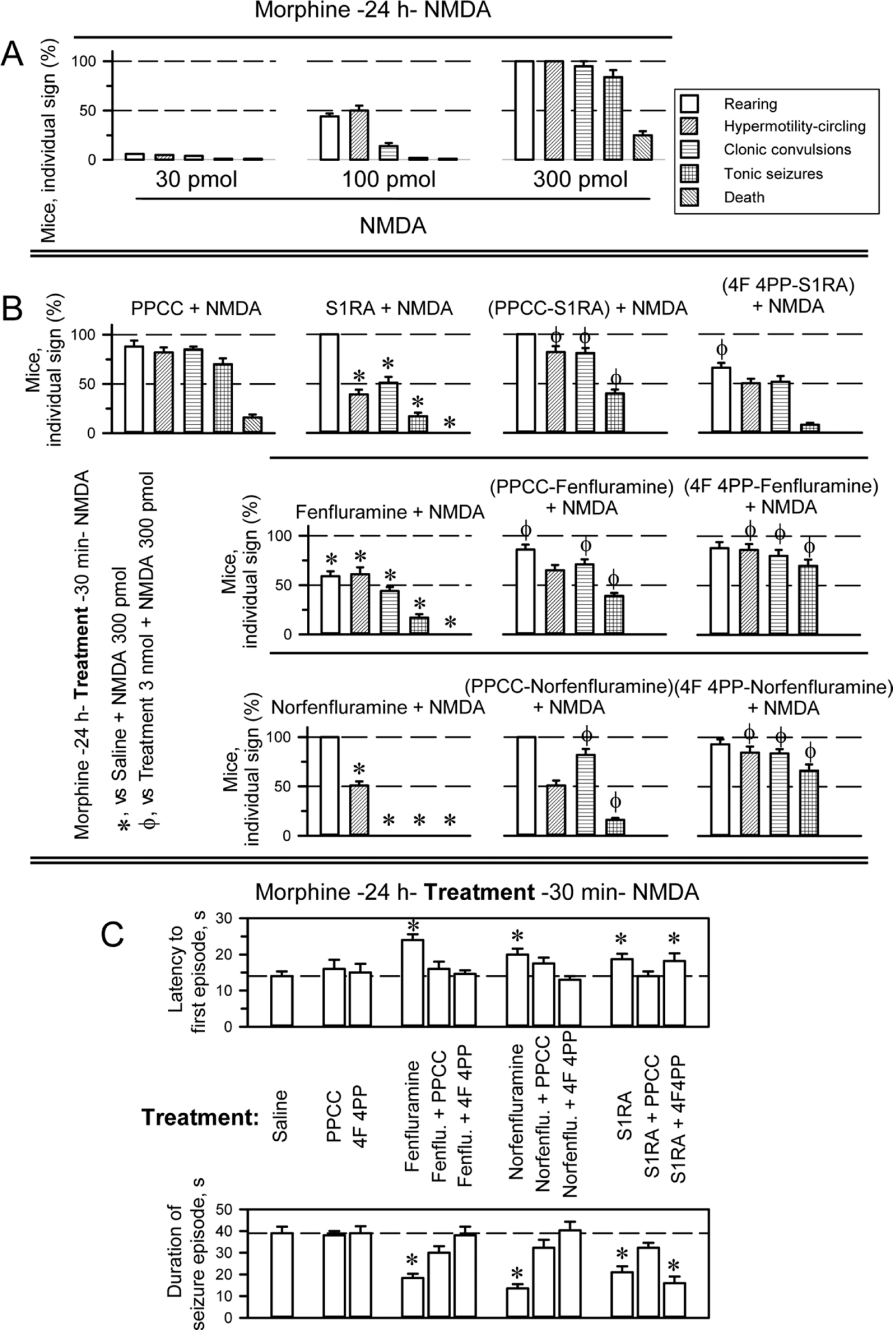
Anticonvulsant effect of fenfluramine and S1RA in a mouse model of seizures induced by NMDAR over activation (**A**) Behavioral alterations produced by icv administration of NMDA to mice pretreated (–24 h) with the opioid agonist morphine (10 nmol, icv). (**B**) Effects of S1RA, fenfluramine and norfenfluramine (3 nmol each, icv) on seizures induced by NMDA (300 pmol, icv) in morphine-primed mice. The drugs were injected icv 30 min before the administration of the NMDA receptor agonist. ^*^Significant difference from the control group, which received morphine and 24 h later NMDA, but saline instead of treatment; ϕ indicates that 3 nmol icv PPCC or 3 nmol icv 4F 4PP prevented the diminishing effects of S1RA/fenfluramine/norfenfluramine on the corresponding NMDA-induced behavioral sign. (**C**) Effects of the treatments on the latency and duration of the seizure episodes. ^*^Significant difference from the control group receiving saline. ANOVA, Dunnett multiple comparisons vs control group, *p* < 0.05. (A–C) Each bar indicates the percentage of mice showing the indicated sign and represents the mean ± SEM of 9 mice.

The selective σ1R ligands S1RA and PPCC at the intracerebroventricular (icv) doses of 3 nmol and 10 nmol, along with fenfluramine and norfenfluramine at the icv dose range of 10–50 nmol and 4F 4PP at the icv dose range of 3–20 nmol, did not cause significant alterations in the motor activity of mice, as determined in the open field [47]. The anticonvulsant activity of fenfluramine was compared to that of the selective σ1R antagonist S1RA in an animal model of seizures induced by icv administration of NMDA [48]. Morphine, acting at MORs, primes NMDARs via the PKC/Src pathway, greatly enhancing the response of NMDARs to direct activators. Thus, icv injection of 10 nmol morphine reduced, from nmol to pmol, the minimum NMDA dose necessary to induce tonic convulsions in 95% of the mice [49, 48]. In pilot assays, we determined that in the absence of morphine, mice treated with 3 nmol NMDA exhibited a convulsive syndrome comparable to that observed in morphine-primed mice treated with 300 pmol NMDA. Although we only tested a few mice in the high-NMDA-dose paradigm, the drugs under study produced effects comparable to those observed with the low NMDA dose in morphine primed mice. Thus, in our paradigm, to reduce the dose of NMDA, we primed the mice with morphine 24 h before icv administration of 300 pmol NMDA in combination with the potential anticonvulsant drugs. With this procedure, practically all the mice exhibited a series of anomalous behaviors such as compulsive rearing, wild running (hypermobility and circling), clonic convulsions, tonic seizures, and, in approximately 15–20% of the animals, death (Figure 2A and Supplementary Videos 1–5).

While 3 nmol of the σ1R agonist PPCC did not significantly alter the behavioral syndrome evoked by NMDA, 3 nmol S1RA reduced the incidence of wild running and clonic convulsions by approximately half, with tonic seizures being present in only 20% of the mice and the total absence of death. PPCC counteracted the activity of S1RA on the NMDA-induced convulsive syndrome, indicating that S1RA indeed produced these beneficial effects by acting at σ1Rs (Figure 2B). Fenfluramine and norfenfluramine used at 3 nmol reduced the appearance of most NMDA-induced signs. Fenfluramine was more efficacious at reducing rearing, while norfenfluramine outperformed its parent compound in abolishing clonic convulsions and tonic seizures. Both compounds protected the mice from NMDA-provoked death. In the presence of the σ1R agonist PPCC, the capacity of fenfluramine and norfenfluramine to diminish certain signs of the NMDA syndrome was impaired. Notably, although icv administration of the serotonin 5HT2AR antagonist 4F 4PP at 3 nmol hardly affected S1RA performance in this paradigm (increased control over rearing behavior), it antagonized the effects of fenfluramine and norfenfluramine (Figure 2B). However, neither PPCC nor 4F 4PP modified the latency to the first convulsive episode or its duration. S1RA, fenfluramine and norfenfluramine increased this latency and reduced the duration of the seizure episode, and PPCC antagonized the effects of the three compounds. Moreover, 4F 4PP antagonized the effects of fenfluramine and norfenfluramine but not S1RA (Figure 2C). These data indicate that the agonist activity of fenfluramine and norfenfluramine on 5HT2ARs is essential to their anticonvulsive effects and that antagonism at σ1Rs increases their efficacy to inhibit NMDAR overactivity.

**Figure 3:**
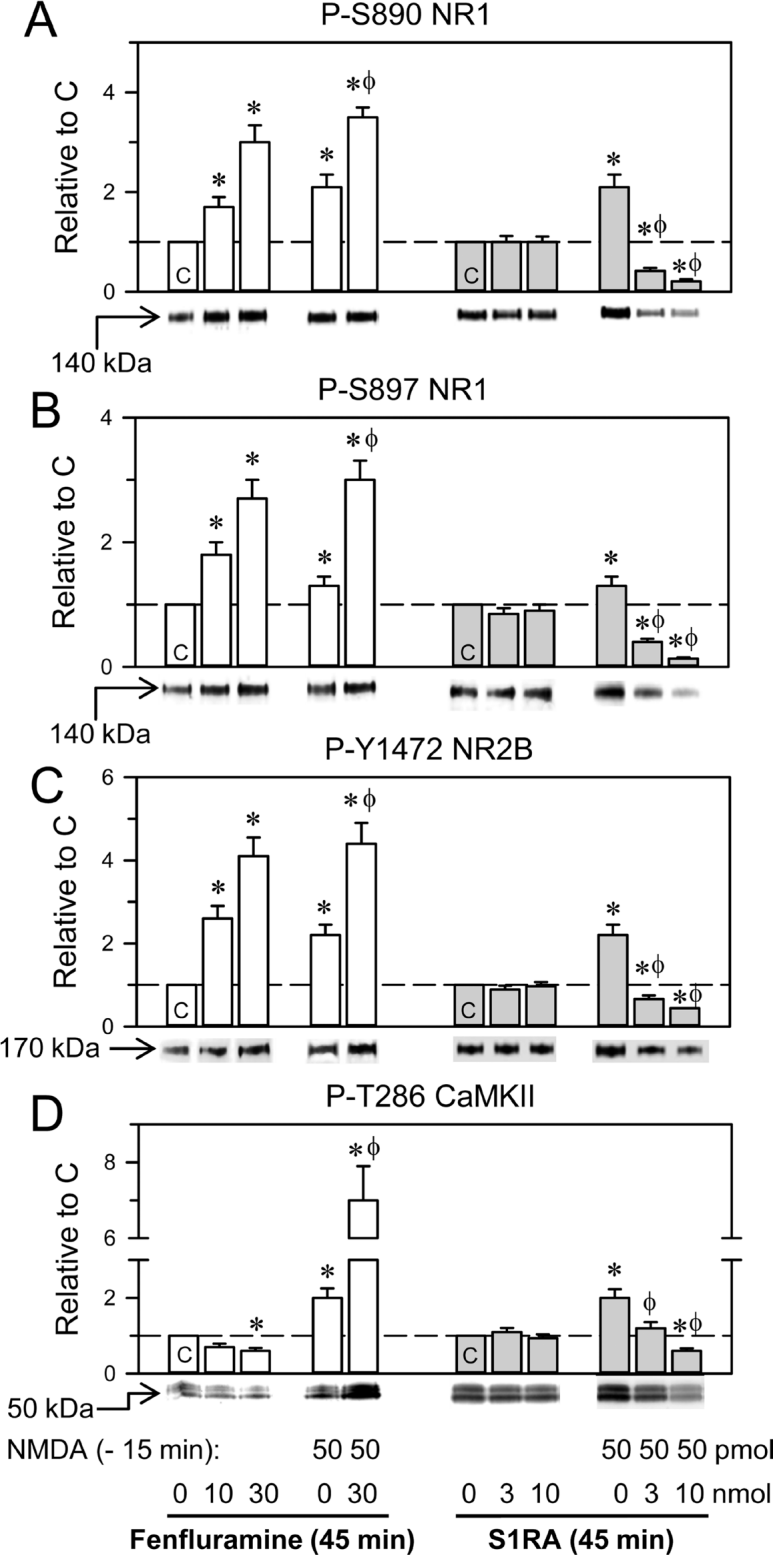
Effects of fenfluramine and S1RA on phosphorylation associated with the NMDAR/CaMKII pathway The mice received icv fenfluramine or S1RA at the doses indicated, and after 1 h, the animals were euthanized and their cerebral cortices obtained for *ex vivo* examination. An activator of NMDARs was administered 1 h before euthanasia, 15 min before S1RA or fenfluramine. The effects of these treatments on NMDAR-related molecular changes were determined by western blot analysis of cortical synaptosomal membranes, (**A**–**C**) Regulatory phosphorylations on NMDAR NR1 subunits (S: serine), and NR2B subunits (Y: tyrosine). (**D**) Phosphorylation of threonine (T) 286 CaMKII. Immunosignals (average optical density of the pixels within the object area/mm^2^) are expressed as the change relative to the control group (C), which is assigned an arbitrary value of 1. Actin was used as a loading control, and the immunosignals did not differ by more than 10% (Supplementary Figure 2). Each bar represents the mean ± SEM of 6 mice. ^*^Significant difference with respect to the control group (C), which received only saline; ϕ significant effect of S1RA or fenfluramine on the activity of NMDA; ANOVA, Dunnett multiple comparisons vs control group, *p* < 0.05.

In *ex vivo* assays, we addressed the effects of the icv administration of fenfluramine and S1RA on a series of parameters related to NMDAR activity. While, fenfluramine in the dose range of 10-30 nmol enhanced the phosphorylation levels of certain residues of NMDAR subunits, such as S890 NR1 (PKC site), S897 NR1 (PKA site) and Y1472 NR2B (Src site), S1RA 3 nmol or 10 nmol did not produce significant changes in these parameters (Figure 3A–3C). NMDA at 300 pmol and higher doses promoted reductions in plasma membrane NMDAR subunits and CaMKII autophosphorylation, alterations that diminished with treatments that prevent NMDAR overactivation. For the sake of a more direct comparison of the molecular effects promoted by the treatments under study, in these assays, we reduced the dose of NMDA to 50 pmol, which produced no significant changes in plasma membrane NMDARs. Under these circumstances, the phosphorylation levels of the NMDAR subunits and CaMKII could be better ascertained. Thus, the administration of 50 pmol NMDA increased phosphorylation at S897 NR1 by approximately 30% and increased phospho- (P-) S890 NR1 and P-Y1472 NR2B 2-fold (Figure 3A–3C). Fenfluramine, given 15 min after NMDA, further augmented the phosphorylation of these sites. In this protocol, S1RA significantly diminished the NMDA-evoked phosphorylation of these subunits (Figure 3A–3C).

CaMKII is an effector of NMDARs, and the calcium- and CaM-dependent activation of CaMKII autophosphorylation at T286 is a marker of the onset of NMDAR activity. While NMDA promoted P-T286 CaMKII, fenfluramine and S1RA did not change or slightly diminished the basal levels of this phosphorylation. On the other hand, S1RA diminished the NMDA-induced phosphorylation of CaMKII (Figure 3D); however, fenfluramine injected 15 min or 1 h after NMDA enhanced or diminished NMDA-promoted CaMKII autophosphorylation, respectively. Both opposite effects of fenfluramine were dampened by 4F 4PP, an antagonist on 5HT2ARs (Figure 3 and Supplementary Figure 1). Thus, at the shorter time interval, fenfluramine via 5HT2Rs collaborated with NMDA in the activation of NMDARs; however, after NMDA had promoted a substantial NMDAR activity, fenfluramine, similar to S1RA, disrupted the σ1R-NR1 association, which likely allowed the inhibitory binding of calcium-activated CaM to the NMDARs [36].

**Figure 4:**
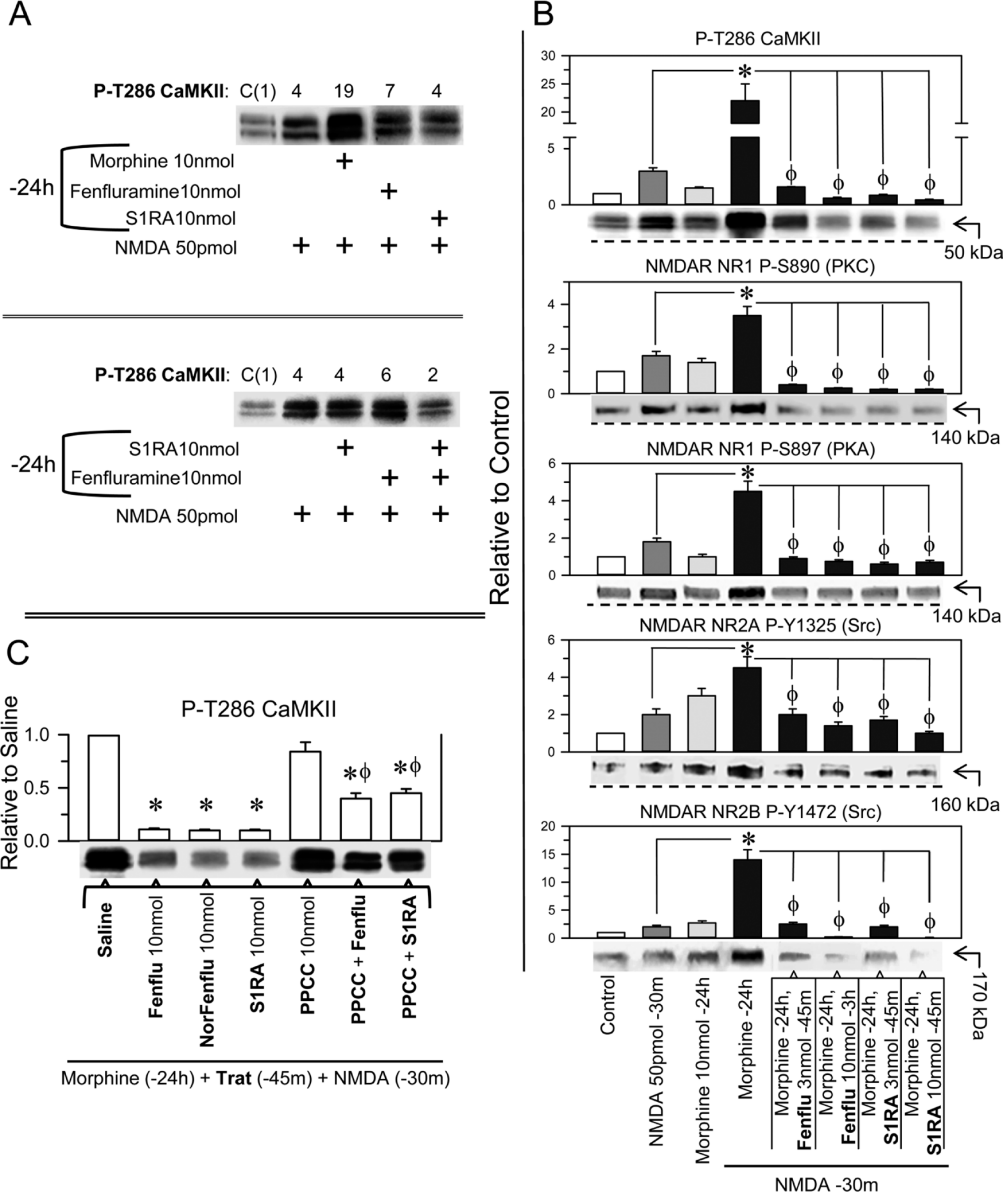
Effect of fenfluramine and S1RA on the overactivity of NMDARs promoted by NMDA in morphine-primed mice (**A**) Morphine-primed mice exhibited a high level of CaMKII auto phosphorylation in response to NMDA. This effect was not evoked by fenfluramine or S1RA, which instead diminished P-T286 CaMKII levels in those morphine and NMDA-treated mice. The assay was repeated at least twice. The immunosignals are expressed relative to the control (C, value of 1), which received saline instead of the treatments indicated. (**B**) Effect of the different treatments on NMDAR phosphorylation and on P-T286 CaMKII. All substances were given icv, and the mice were killed at the post-drug intervals indicated, *e.g*., 30 min after NMDA and 24 h after morphine. The combined treatments started with morphine, and 24 h later, fenfluramine or S1RA was administered 45 m before NMDA. ^*^ Significant difference with respect to the group that received only morphine; ϕ significant difference respect to the morphine-primed group that received only NMDA 24 h later; ANOVA, Dunnett multiple comparisons vs control group, *p* < 0.05. (**C**) Effect of the σ1R agonist PPCC on the diminished effect of fenfluramine and S1RA on CaMKII hyper phosphorylation evoked by NMDA. The mice were primed with morphine, and 24 h later, the effects of the different drugs on P-T286 CaMKII were evaluated. ^*^Significant difference with respect to the morphine-primed group that received saline instead of the drug and NMDA 24 h later; ϕ significant difference with respect to the group that received fenfluramine or S1RA but not PPCC; ANOVA, Dunnett multiple comparisons vs control group, *p* < 0.05. (A–C) Each bar represents the mean ± SEM of 6 mice. Details as in Figure 3 and Supplementary Figure 3.

In the NMDAR overactivation paradigm, the mice received a single icv dose of 10 nmol morphine, and 24 h later, when the morphine had been cleared, we addressed the effect of fenfluramine and S1RA on NMDA-activated NMDARs. In the morphine-primed animals, NMDA greatly stimulated the NMDAR/CaMKII signaling pathway, and the levels of P-T286 CaMKII increased approximately 6-fold with respect to the mice not treated with the opioid (Figure 4A and 4B). Unlike morphine, fenfluramine and S1RA icv injections 24 h before NMDA did not significantly enhance the autophosphorylation of CaMKII (Figure 4A). In morphine-primed mice, NMDA also enhanced the levels of P-S890 and P-S897 NR1, P-Y1325 NR2A and P-Y1472 NR2B. In this protocol, the administration of fenfluramine and S1RA 45 min before NMDA diminished NMDAR-activating phosphorylations and reduced NMDAR-mediated calcium influxes, as determined through the reduction of P-T286 CaMKII (Figure 4B). Notably, the σ1R agonist PPCC diminished the capacity of S1RA and fenfluramine to inhibit NMDA-mediated activation of CaMKII in morphine-primed mice (Figure 4C). Thus, fenfluramine given 45 min before NMDA administration prevented NMDAR overactivation through its antagonism at σ1Rs (Figure 4).

**Figure 5:**
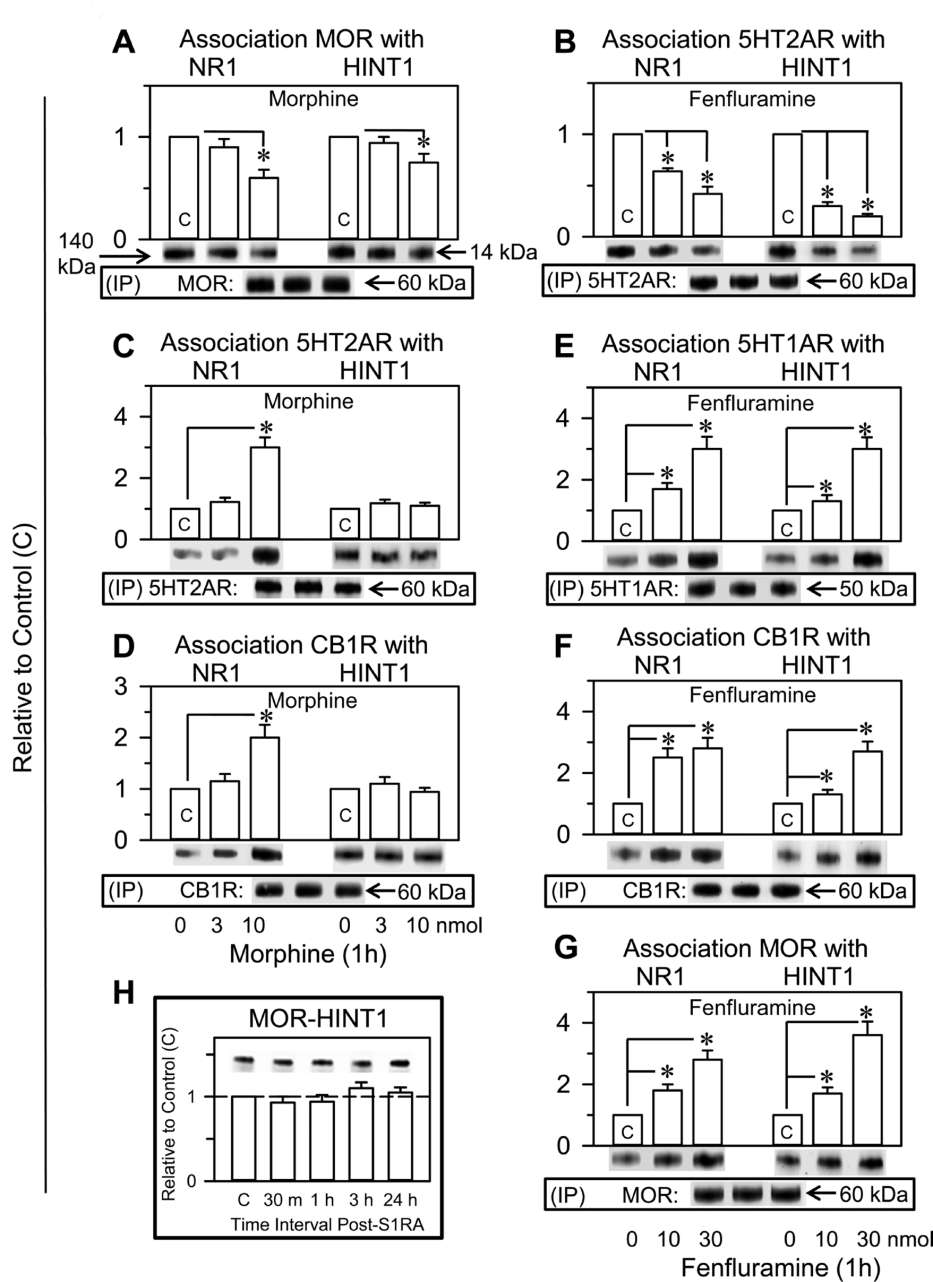
Fenfluramine transfers control of NMDARs and HINT1 proteins to other GPCRs such as 5HT1ARs, CB1Rs or MORs. Comparative study with morphine and S1RA Fenfluramine (**B, E, F, G**) and morphine (**A, C, D**) were both icv injected into the mice at 10 nmol; S1RA (**H**) was icv injected at 3 nmol. At the intervals indicated, the animals were euthanized to obtain the synaptosomal cortical fraction. The GPCRs were immunoprecipitated (IP), and the associated NR1 subunits and HINT1 protein were assessed by western blot analysis. Each bar represents the mean ± SEM of 8 mice. ^*^Significant difference with respect to the control group, which received saline instead of the drug; ANOVA, Dunnett multiple comparisons vs control group, *p* < 0.05. Details are in the Methods section.

The association of certain GPCRs, such as MOR and CB1R, with NMDARs is regulated by HINT1-σ1R complexes (see Introduction). In this context, the effects of fenfluramine and morphine on GPCR-NMDAR associations were addressed. In 1 h, the icv administration of 10 nmol morphine reduced the association of MORs with NR1 subunits by approximately 40% and that with HINT1 proteins by 20 % (Figure 5A). Furthermore, 10 nmol fenfluramine reduced the association of 5HT2ARs with NR1 and HINT1 proteins by approximately 30% and 75% respectively (Figure 5B).

Morphine was observed to increase the association of other GPCRs, such as 5HT2ARs and CB1Rs, with NR1 subunits but not with HINT1 proteins (Figure 5C and 5D). In contrast, fenfluramine enhanced the association of 5HT1ARs, CB1Rs and MORs not only with NR1 subunits but also with HINT1 proteins (Figure 5E–5G), suggesting that, on activation of 5HT2Rs, its antagonism at σ1Rs collaborated to transfer the HINT1 proteins. In the absence of pharmacological activation of GPCRs, the selective σ1R antagonist S1RA did not increase the association of resting MORs with HINT1 proteins (Figure 5H).

## DISCUSSION

An excess of glutamatergic neurotransmission, particularly at NMDARs, is essential to the clinical manifestations of epilepsy [1–3], and drugs such as fenfluramine reduce NMDAR overactivity without triggering the undesirable side effects caused by direct antagonists. Our study indicated that the potent anticonvulsant effect of fenfluramine is the result of its complex pharmacological profile, which includes serotonin GPCRs [19, 18], σ1Rs, and probably, serotonin metabolism [50, 51]. Thus, fenfluramine takes advantage of the utility of σ1R antagonists [41, 40, 38] and of serotonergic substances in the control of seizures and convulsive episodes [52]. This idea could also apply to monoamine reuptake inhibitors and specific serotonin reuptake inhibitors, such as fluoxetine and citalopram, that exhibit anticonvulsant activity [53] and display activity at σ1Rs [54].

Different GPCRs can promote NMDAR activity through PLCβ- and PKC-mediated activation of Src, *e.g*., MORs [55] and 5HT2A/CRs [35], while others, such as 5HT1ARs [23, 56] and CB1Rs [57] reduce NMDAR function. In the brain, fenfluramine displays agonist activity on 5HT2ARs and 5HT2CRs [19, 18], and both serotoninergic GPCRs stimulate the activation of NMDARs [35]; however, its serotonergic activity, working together with its concomitant antagonism at σ1Rs, caused the negative control of NMDARs. The activity of fenfluramine on 5HT2CRs was not directly evaluated in our study; however, this drug may also promote the dampening of NMDAR function through σ1Rs and this serotonin receptor. Because serotonergic 5HT2CRs are actually considered a potential therapeutic target for designer antiepileptic drugs [53], whether the successful drugs display activity at the σ1R should be determined.

The mixed activity of fenfluramine resulted in molecular changes that differed from those promoted by just a GPCR agonist. In the absence, of a relevant pool of primed NMDARs, fenfluramine increased the phosphorylation of several specific residues in NMDAR NR1 and NR2 subunits and diminished the association between 5HT2ARs and NMDAR NR1 subunits. These effects satisfactorily compared with those promoted by morphine-activated MORs, which are known to recruit the function of NMDARs [36]. However, morphine, but not fenfluramine, greatly increased the activating autophosphorylation of T286 CaMKII [36], a useful marker of NMDAR activity onset. As a result, fenfluramine augmented the activating phosphorylation of NMDARs without promoting the levels of NMDAR activity attained by morphine. The opioid agonist displays an affinity (K_D_) at MORs in the low nM range (1 to 3 nM) [58], and a dose of 10 nmol efficaciously activates the NMDAR/CaMKII pathway, as determined by P-T286 CaMKII [36]. In the brain, the K_D_ of norfenfluramine and fenfluramine on 5HT2Rs is approximately 200 nM and 2 μM, respectively [18]. Notwithstanding, fenfluramine at 10 nmol failed to activate CaMKII, but even its lower dose of 3 nmol prevented the response of the morphine-primed NMDARs to their agonist NMDA.

The observations mentioned above indicate that the fenfluramine inhibition of MOR-primed NMDARs cannot be produced merely by the activation of 5HT2A/2C receptors, which, similar to MORs, are coupled to the activation of NMDARs [35, 31]. The antagonism displayed by fenfluramine on σ1Rs accounts for the inhibitory effect of the NMDAR function. The association of σ1Rs with GPCR-primed NMDARs has a regulatory function and is essential to the anticonvulsant effects herein described. Thus, in its binding to the cytosolic C terminal region of NR1 subunits, the σ1R blocks the access of calcium-activated CaM to reduce the open probability of the NMDAR channel and, consequently, to inhibit calcium influxes [45]. At the neuronal plasma membrane, σ1R agonists promote, and antagonists diminish, σ1R-NR1 associations [36, 37]. Indeed, the σ1R antagonist S1RA did not promote the NMDAR/CaMKII pathway and diminished the activating phosphorylation of NMDARs independent of their degree of priming/activation. S1RA disrupted the σ1R-NR1 interactions with an ED_50_ of 1.5 pM, while fenfluramine and norfenfluramine promoted this effect at 170 pM and 50 pM, respectively. Therefore, fenfluramine may act through 5HT2A/CRs and σ1Rs to achieve its efficacious control of the NMDAR function.

In the absence of morphine priming, 50 pmol NMDA moderately increased the PKC-, PKA- and Src-mediated phosphorylation of NMDAR subunits, as well as T286 CaMKII autophosphorylation. Whereas, in this protocol, S1RA administered shortly after NMDA diminished the markers of the NMDAR/CaMKII pathway, fenfluramine further increased their presence, P-T286 CaMKII included. When fenfluramine was injected after NMDA had already enhanced the NMDAR function, this drug decreased the activity of NMDARs, as determined through the levels of autophosphorylation of CaMKII. In the scenario of moderated NMDAR activity, the agonist effect of fenfluramine on 5HT2Rs prevailed over its antagonism at σ1Rs and contributed to NMDAR activity. However, in a situation of NMDAR overactivation, such as that attained in morphine-primed mice, fenfluramine diminished the responsiveness of NMDARs. These observations suggest that fenfluramine more effectively disrupted the binding of σ1Rs to the GPCR-freed overactivated NMDARs, enabling calcium-activated CaM to inhibit NMDAR function.

Notably, fenfluramine greatly reduced the association of 5HT2ARs with HINT1 proteins and with NMDAR NR1 subunits. The activity of fenfluramine and its nor-metabolite on 5HT2ARs may combine with their antagonism at σ1Rs to promote the diminishing effects on the 5HT2AR-NR1 and particularly on the 5HT2AR-HINT1 associations. In fact, in the presence of σ1R antagonists, the activation of GPCRs transfers HINT1 proteins, instead of σ1Rs, from the GPCRs towards the NMDAR NR1 subunits [37, 44]. The fenfluramine-induced transfer of HINT1 proteins is of relevance to the restrain of the NMDAR activity. In the plasma membrane, HINT1 can bind to cytosolic residues in GPCRs and NMDAR NR1 subunits [59, 60]. In the GPCR-HINT1-σ1R-NMDAR complex, HINT1 binds to the GPCR, the NMDAR remains silent, and the σ1R prevents HINT1 from binding to NR1 subunits. The activation of GPCRs, such as MORs, causes the separation of the primed NMDARs, which then allow calcium to permeate and display a high affinity toward σ1Rs. Thus, the MOR-HINT1 complex remains separated from the primed NMDAR that carries the σ1R to prevent the inhibitory binding of Ca^2+^-CaM [36]. However, if antagonists reach σ1Rs when in the MOR-HINT1-σ1R-NMDAR complex, the antagonists prevent σ1Rs from binding to NMDAR NR1 subunits, and thus, MOR activation transfers HINT1 to the NR1 subunit, and this has been observed for morphine and S1RA [36].

The transfer of HINT1 proteins from agonist-activated GPCRs to NMDARs impairs the capacity of these GPCRs to recruit additional glutamatergic activity and accelerates the inactivation of HINT1-bound and active NMDARs [37]. This is because, the binding of HINT1 to NR1 subunits is substantially different from that of the σ1R, and HINT1 does not prevent Ca^2+^-CaM from binding and inhibiting NMDAR function [36]. On the other hand, GPCRs require HINT1 proteins to couple to silent NMDARs [61], and depletion of HINT1 proteins prevents GPCRs from binding to NMDARs and to promote their function. Indeed, in HINT1^-/-^ mice, MOR-acting opioids do not stimulate NMDAR/CaMKII activity [62]. Interestingly, NMDARs when coupled with silent GPCRs are refractory to their activators [56], and thus, increases in NMDAR-GPCR association diminish the pool of NMDARs available for direct activation. Morphine by acting at MORs increased the association of other GPCRs with NR1 subunits. Fenfluramine induced a greater transfer of not only NR1 subunits but also HINT1 proteins toward GPCRs inhibitory of NMDAR function, such as 5HT1ARs and CB1Rs. This is an effect that may contribute to the negative control of fenfluramine on NMDAR function.

**Figure 6:**
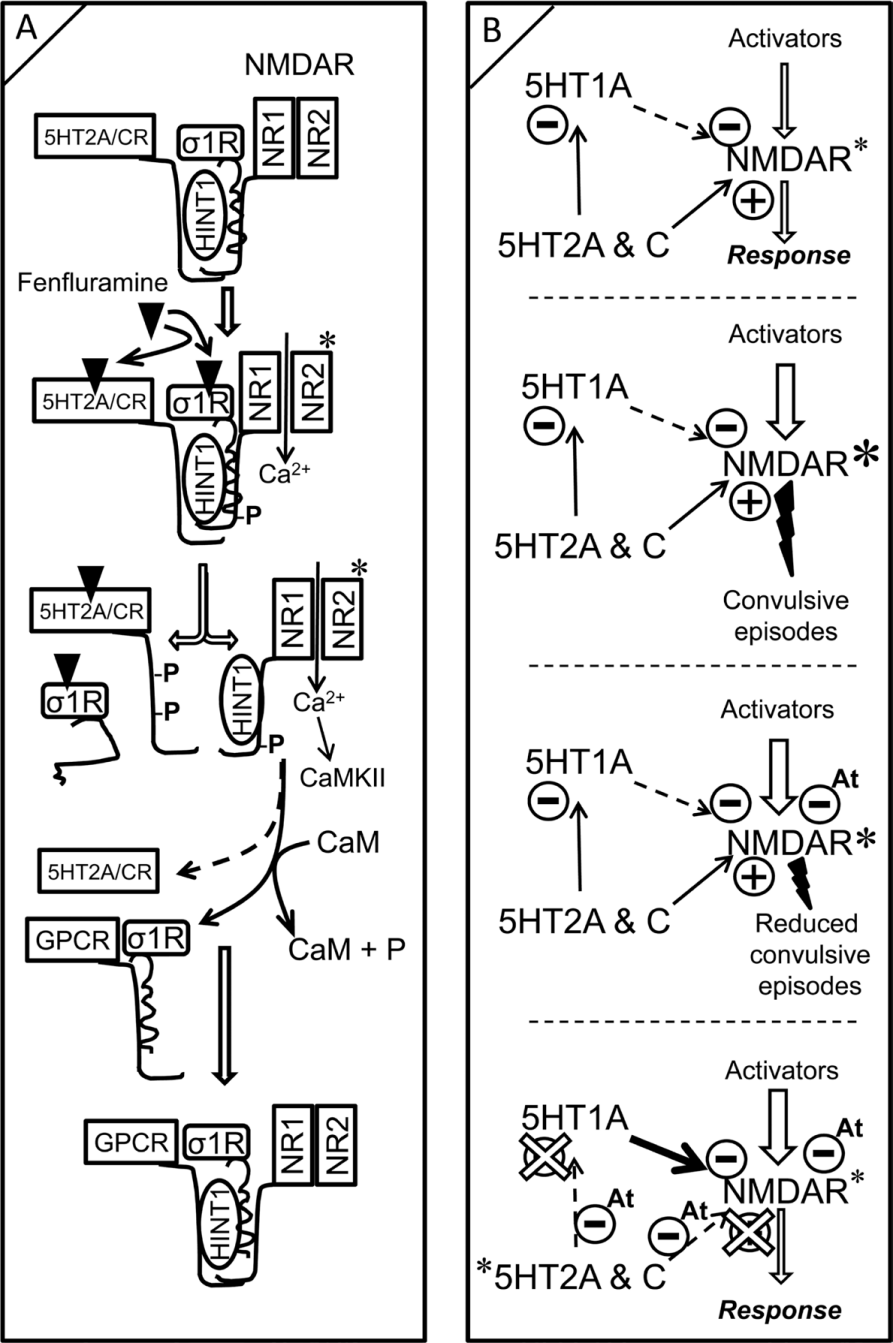
Modeling fenfluramine activity at 5HT2A/C and σ1 receptors. Influence on negative control of NMDAR function by the 5HT1A receptor (**A**) A certain pool of 5HT2A/C receptors is complexed with NMDARs via HINT1 proteins and σ1Rs. Fenfluramine binds and activates 5HT2A/C receptors, promoting PKC/Src priming of the coupled NMDARs. The binding of antagonists such as fenfluramine to σ1Rs prevents σ1Rs from binding to NMDARs [37], enabling HINT1 transfer from the GPCR to NMDAR NR1 subunits. The phosphorylation of 5HT2A/CR cytosolic residues inhibits its re-association with HINT1 to recruit further NMDAR activity. Because HINT1 allows CaM to access NR1 subunits [36], the inactive HINT1-NMDARs can now couple with other GPCRS such as CB1Rs and 5HT1ARs. (**B**) While 5HT1ARs inhibit NMDAR activity, 5HT2A/CRs promote NMDAR function directly and furthermore, promote it indirectly through the negative control of 5HT1AR signaling. Exaggerated activation of NMDARs may promote seizures. Antagonists of σ1Rs (At) remove σ1Rs from NR1 subunits and enable negative control by CaM; the incidence of convulsive episodes diminishes. The combined activity of fenfluramine as agonist at 5HT2R and antagonist at σ1R uncouples 5HT2Rs from positive control of NMDAR function and increases the inhibitory coupling of GPCRs, such as the 5HT1AR, to NMDAR function. In addition, fenfluramine, as a σ1R antagonist, facilitates CaM inhibition of already primed and GPCR-freed NMDARs. Fenfluramine, by triggering these mechanisms, efficaciously reduces the incidence of epileptogenic episodes. A and B, ^*^indicates activation; B, ^*^indicates higher activity than^*^.

The mechanisms triggered by fenfluramine to diminish NMDAR activity may be as follows (Figure 6A): fenfluramine activates 5HT2ARs and, probably 5HT2CRs, and primes NMDARs to promote their release and activation. When NMDARs reach a high level of activity, such as that seen in morphine-primed mice, fenfluramine prevents σ1Rs from binding to NR1 subunits. The absence of σ1Rs facilitates the transfer of HINT1 proteins from the fenfluramine-activated 5HT2Rs to the NR1 subunits [36]. Depletion of HINT1 proteins at 5HT2Rs reduces the capacity of fenfluramine to recruit more NMDAR activity and stops the 5HT2R-mediated activation of NMDARs. The calcium-activated CaM binds to and inactivates 5HT2R-freed NMDAR-HINT1 complexes, thus counteracting the initial activation of NMDARs promoted by fenfluramine at 5HT2Rs. Moreover, those NMDARs inactivated by calcium-CaM now associate with GPCRs such as CB1Rs and 5HT1ARs, and this is relevant to the control of NMDAR activity.

Indeed, the endocannabinoid system sequesters silent NMDARs via CB1Rs and diminishes the pool of NMDARs that are ready for activation [63, 21, 64], and fenfluramine indirectly promotes this activity. 5HT1ARs, similar to CB1Rs, are also coupled to the inhibition of NMDAR ionic fluxes [23]; however, 5HT2A/CRs counteract the activity of 5HT1ARs and thereby contribute to the NMDAR function [35]. Interestingly, fenfluramine increases the coupling of 5HT1ARs with HINT1 and NMDARs, and thus, it may indirectly promote the inhibitory effect of 5HT1ARs on NMDAR function (Figure 6B). Because fenfluramine and its metabolites impair the reuptake of serotonin by acting on the vesicular storage of the neurotransmitter [50, 51], the increased availability of serotonin may facilitate 5HT1AR-mediated control of NMDAR activity.

In a putative model of Dravet syndrome in zebrafish, drugs with serotonergic activity reduce the incidence of epileptic seizures [65, 66]. Fenfluramine causes these effects through its action on the zebrafish orthologues of serotonin 5HT1D and 5HT2C receptors but not 5HT2A receptors. In this model, agonists and antagonists of the zebrafish σ1R promoted similar reductions in convulsive phenotypes, suggesting the possibility that this ligand-regulated chaperone participates in the beneficial activity of fenfluramine [67]. In our rodent model of seizures promoted by NMDAR overactivation, the beneficial effects of fenfluramine and norfenfluramine were prevented by the selective 5HT2AR antagonist 4F 4PP and only partially by the σ1R agonist PPCC. Thus, in mammals, the activity of fenfluramine and its metabolites on σ1Rs and, consequently, the enhancement of CB1R and 5HT1AR control over NMDAR activity are downstream of the 5HT2AR.

The comparison of zebrafish findings with our data is complicated because whether zebrafish serotonin receptors regulate signaling pathways comparable to those in the mammalian brain and exert similar regulation of NMDAR function has not been determined. As aforementioned, in the rodent brain, fenfluramine may act as an agonist on other serotonin receptors, such as 5HT2CR, to contribute to the control of seizures related to *SCN1A* sodium channel dysfunction. Notwithstanding, agents that block NMDARs are effective in developmental animal models of epilepsy [68] and clinically relevant in the treatment of childhood epileptic encephalopathies such as Lennox-Gastaut syndrome [69]. Consequently, the NMDAR seems to play an essential role in the expression of these convulsive syndromes.

Thus, the experimental MOR priming of NMDARs may reasonably represent situations of excitotoxic risk such as those promoted by epileptogenic foci in mammals. The serotonin 5HT2Rs and σ1 receptors appear to be essential for fenfluramine to promote its anticonvulsive effects. Our study provides molecular support for the use of mixed drugs such as fenfluramine to diminish the incidence and extent of seizures promoted by the overactivity of NMDARs.

## MATERIALS AND METHODS

### Animals

Male albino CD-1 mice (Charles River) were used in the study. The mice were maintained at 22° C on a diurnal 12 h light/dark cycle. All procedures involving mice adhered strictly to the guidelines of the European Community for the Care and Use of Laboratory Animals (Council Directive 86/609/EEC) and Spanish Law (RD53/2013) regulating animal research. Each group consisted of 6 to 9 animals, which were used only once.

### Experimental protocol

Seizures can typically be induced by icv injection of 3 to 10 nmol NMDA into a lateral ventricle of the mice [49, 48]. After mice are primed with morphine, the dose of NMDA needed to induce tonic convulsions in 95% of mice is reduced from nmol to pmol. Thus, in our paradigm, to reduce the dose of NMDA, we administered an icv injection of 10 nmol morphine to mice 24 h before icv administration of 300 pmol NMDA. The drugs were dissolved in sterile saline and injected in a volume of 4 μL into a lateral ventricle using a procedure similar to that described by others [48]. This pmol dose of NMDA was selected because it was the minimal dose that reliably induced the appearance of tonic seizures in at least 80% of treated mice. Immediately after injection animals were placed in a transparent box (20×20×30 cm) and were observed for a period of 3 min. The seizure activity consisted of a mild myoclonic phase (immobility, mouth and facial movements, tail extension, circling), rearing (violent movements of the whole body, rearing), wild running (episodes of running with explosive jumps), and clonic convulsions (characterized by rigidity of the whole body including limb flexion/extension), followed by continuous/repetitive seizure activity (tonic seizures) and, in approximately 15–20% of the animals, death. The episode typically began a few seconds after injection and evolved to its maximal intensity in less than 1 min. The results are expressed as the percentage of mice exhibiting the aforementioned signs and the mean latencies of the first body clonus.

The compounds used were: morphine sulfate (Merck, Darmstadt, Germany); *N*-methyl-D-aspartic acid (NMDA) (#0114, Tocris); (+)-fenfluramine hydrochloride (#2695, Tocris); (+)-norfenfluramine hydrochloride (#N3288, Sigma-Aldrich); S1RA (#16279, Cayman Chemical); (±)-PPCC oxalate (#3870, Tocris); progesterone (#P7556, Sigma-Aldrich), 4F 4PP oxalate (#0523, Tocris). Test drugs were dissolved in saline except PPCC and 4F 4PP that were prepared in 1:1:18 (v/v/v) mixture of ethanol:Kolliphor EL (#C5135, Sigma-Aldrich): physiological saline, and icv injected 30 min before NMDA administration. Doses and intervals were selected based on previous studies [36, 37] and pilot assays. The motor performance of mice administered the solvents used was identical to non-injected animals.

### Expression of recombinant proteins

The coding region of murine full-length (1-223) σ1R (AF004927), and the C-terminal region of the glutamate receptor NMDAR1 (NM_008169) (residues 834-938), were all amplified by RT-PCR using total RNA isolated from mouse brains as the template. Specific primers containing an upstream Sgf I and a downstream Pme I restriction site were used, as described previously [36]. The PCR products were cloned downstream of the GST coding sequence and the TEV protease site. The sequenced proteins were identical to the GenBank™ sequences. The vector was introduced into E. coli BL21 (KRX #L3002, Promega, Madrid, Spain), and clones were selected on solid medium containing ampicillin. After 3 h induction at room temperature (1 mM IPTG and 0.1% Rhamnose), the cells were collected by centrifugation, and the pellets were maintained at –80° C.

The purification of GST fusion proteins was done under native conditions on GStrap FF columns (GE#17-5130-01, Healthcare, Barcelona, Spain) and when necessary the fusion proteins retained were cleaved on the column with ProTEV protease (Promega, #V605A) and further purification was achieved by high-resolution ion exchange (Enrich Q, BioRad #780-0001) or electroelution of the corresponding gel band (GE 200, Hoefer Scientific Instruments, San Francisco, CA, USA). The sequences were confirmed through automated capillary sequencing.

### *In vitro* interactions between recombinant proteins: pull-down of recombinant proteins, effect of drugs on σ1R-NR1 interactions

Having demonstrated that the σ1R does not bind to GST (Z02039; GenScript Co., Piscataway, NJ) [44], we determined the association of GST-free σ1R with GST-tagged NR1 C-terminal sequence C0–C1–C2. The NR1 C-terminal sequence was immobilized through covalent attachment to NHS-activated Sepharose 4 fast flow (FF) (GE#17-0906-01) according to the manufacturer's instructions. The recombinant σ1R (100 nM) was incubated either with NHS-blocked Sepharose 4FF (negative control) or with the immobilized NR1 protein in 200 μL of a buffer containing 50 mM Tris-HCl, pH 7.4 and 0.2% CHAPS in the presence of 2.5 mM of calcium chloride. In pilot assays we determined that after 20 min of incubation the NR1-σ1R association was maximal, and that this period of time was also sufficient for the drugs to promote stable changes in their association. Thus, the samples were mixed by rotation for 20 min at RT, and σ1R-bound to NR1-Sepharose 4FF was recovered by centrifugation and three cycles of washing. The agarose attached NR1-σ1R complexes were incubated in the presence of increasing concentrations of the drugs under study for 20 min with rotation at room temperature in 300 μL of 50 mM Tris-HCl, pH 7.5, 2.5 mM CaCl_2_, and 0.2% CHAPS. Agarose pellets containing the bound proteins were obtained by centrifugation, washed thrice, solubilized in 2x Laemmli buffer, and the presence of σ1Rs was addressed by Western blotting.

### Immunoprecipitation and Western blotting

Cerebral cortices were collected and homogenized in 10 volumes of 25 mM Tris-HCl pH 7.4, and 0.32 M sucrose supplemented with a phosphatase inhibitor mixture (P2850; Sigma), H89 (B1427; Sigma), and a protease inhibitor cocktail (P8340; Sigma). The homogenate was centrifuged at 1000 g for 10 min to remove the nuclear fraction. The supernatant (S1) was centrifuged twice at 20,000 g for 20 min to obtain the crude synaptosomal pellet (P2). The final pellet was diluted in Tris buffer supplemented with a mixture of protease inhibitors (0.2 mM phenylmethylsulphonyl fluoride, 2 μg/mL leupeptin, and 0.5 μg/mL aprotinin), then divided into aliquots and processed for protein determinations.

For immunoprecipitation studies and to circumvent interference with signaling proteins attached to the cytosolic regions of the GPCRs, the antibodies used were directed to amino acid sequences in their extracellular domains. The affinity purified IgGs against the extracellular domains of the MOR 2nd external loop (EL) (205-216: MATTKYRQGSID; GenScript Co., Piscataway, NJ, USA), CB1R 1st EL (177-188: DFHVFHRKDSPN; GenScript Co.), 5HT1AR 2nd EL (174-186: GWRTPEDRSNPNE), 5HT2AR Nt (25-38: SRLYPNDFNSRDAN) and the NMDAR NR1 subunit (483-496: KFGTQERVNNSNKK; GenScript Co.) were labeled with biotin (Pierce #21217 and 21339). Pilot assays were performed to optimize the amount of IgG and sample protein needed to precipitate the desired protein in a single run. The Nonidet P-40 solubilized proteins were incubated overnight at 4°C with biotin-conjugated primary antibodies directed against the target protein. The immunocomplexes were recovered and resolved with SDS/polyacrylamide gel electrophoresis (PAGE) in 10 cm × 10 cm × 1.5 mm gel slabs (7–14% total acrylamide concentration, 2.6% bisacrylamide cross-linker concentration). The separated proteins were then transferred onto 0.2 μm polyvinylidene difluoride (PVDF) membranes (162-0176; Bio-Rad, Madrid, Spain) and probed overnight at 6° C with the selected primary antibodies diluted in Tris-buffered saline pH 7.7 (TBS) + 0.05% Tween 20 (TTBS). Those were detected using secondary antibodies conjugated to horseradish peroxidase. The western blot images, antibody binding, were visualized by chemiluminescence (#170-5061; Bio-Rad) and recorded using an ImageQuant™ LAS 500 (GE Healthcare Bio-Sciences AB, Sweden). For each blot two areas of capture were typically selected, the treatment and the loading control. The software automatically calculates the optimal exposure time for each of the specified areas to provide the highest possible signal to enable accurate quantification of the sample. Protein immunosignals, and those of actin, were measured using the area of the strongest signal of each studied group of samples (average optical density of the pixels within the object area/mm2; AlphaEase FC software), the grey values of the means were then normalized within the 8 bit/256 grey levels [(256-computed value)/computed value]. Equal loading was verified and adjusted, if necessary, versus actin. In the GPCR immunoprecipitation studies, the middle region was dedicated to the detection of the GPCR, the upper region for co-precipitated NR1C1 subunits and the lower to HINT1. The secondary antibodies were directed to either the heavy or light IgG chains of the primary antibodies, as needed. Thus, the secondary antibodies also reacted primarily with the separated IgG light chains of the accompanying antibodies used to immunoprecipitation of de target GPCR and provided the loading control for the samples in the gel [70].

### Antibodies

The primary antibodies to detect immunoprecipitated receptors were: anti-MOR Ct aa 387–398 (GenScript Co., Piscataway, NJ), anti-CB1R Nt aa 53-66 (GenScript Co), anti-5HT1AR (#ab64994, Abcam), anti-5HT2AR (#ab16028, Abcam), anti-NMDAR NR1 (#MAB1586, Merck-Millipore), anti-NMDAR NR1 C1 (#MAB5046P, Merck-Millipore). Other primary antibodies used in this study were: anti-σ1R (#42-3300, Invitrogen), anti-CaMKIIpan (#3362, Cell Signaling), anti-CaMKIIα P-T286 (#3361, Cell Signaling), anti-NMDAR NR1 P-S890 (#3381, Cell Signaling), anti-NMDAR NR1 P-S897 (#ABN99, Millipore), anti-NMDAR NR2A P-Y1325 (#ab16646, Abcam), anti-NMDAR NR2B P-Y1472 (#AB5403, Millipore). The anti-HINT1 antibody was raised in rabbits (Immunostep, Spain) against the peptide sequence GYRMVVNEGADGGG (aa 93–106). All primary antibodies were detected using the appropriate horseradish peroxidase-conjugated secondary antibodies.

### Statistical analysis

All graphs and statistical analyses were generated and performed using the Sigmaplot/SigmaStat v.13 package (SPSS Science Software, Erkrath, Germany). Significance was defined as *p* < 0.05. Data were analyzed using one-way ANOVA followed by Dunnett multiple comparisons vs the control group.

## SUPPLEMENTARY MATERIALS FIGURES AND TABLES













## Abbreviations

4F 4PP: 4-(4-Fluorobenzoyl)-1-(4-phenylbutyl)piperidine oxalate
5HT: serotonin
5H1AR: Type 1A serotonin receptor
5HT2AR: Type 2A serotonin receptor
CaM: Calmodulin
CB1R: Type 1 cannabinoid receptor
Fenfluramine: (S)-N-Ethyl-α-methyl-3-(trifluoromethyl) benzeneethanamine hydrochloride
GPCR: G protein-coupled receptor
HINT1: Histidine triad nucleotide-binding protein 1
MOR: Mu opioid receptor
NMDAR: Glutamate *N*-methyl-D-aspartate (NMDA) receptor
Norfenfluramine: S-(+)-α-Methyl-3-(trifluoromethyl)benzeneethanamine hydrochloride
PPCC: (S*,R*)-2-[(4-Hydroxy-4-phenyl-1-piperidinyl)methyl]-1-(4-methylphenyl)- cyclopropanecarboxylic acid methyl ester
σ1R: Type 1 sigma receptor
S1RA: 4-[2-[[5-methyl-1-(2-naphthalenyl)-1H-pyrazol-3-yl]oxy]ethyl]-morpholine
