# Supplementary material for: Fenfluramine diminishes NMDA receptor-mediated seizures via its mixed activity at serotonin 5HT2A and type 1 sigma receptors
Source: Oncotarget. 2018 May 4;9(34):23373–89. doi: 10.18632/oncotarget.25169 (PMC5955088; doi:10.18632/oncotarget.25169)
Supplement: Supplementary file 1 [file oncotarget-09-23373-s001.pdf]

Fenfluramine diminishes NMDA receptor-mediated seizures via its mixed activity at serotonin 5HT2A and type 1 sigma receptors

SUPPLEMENTARY MATERIALS

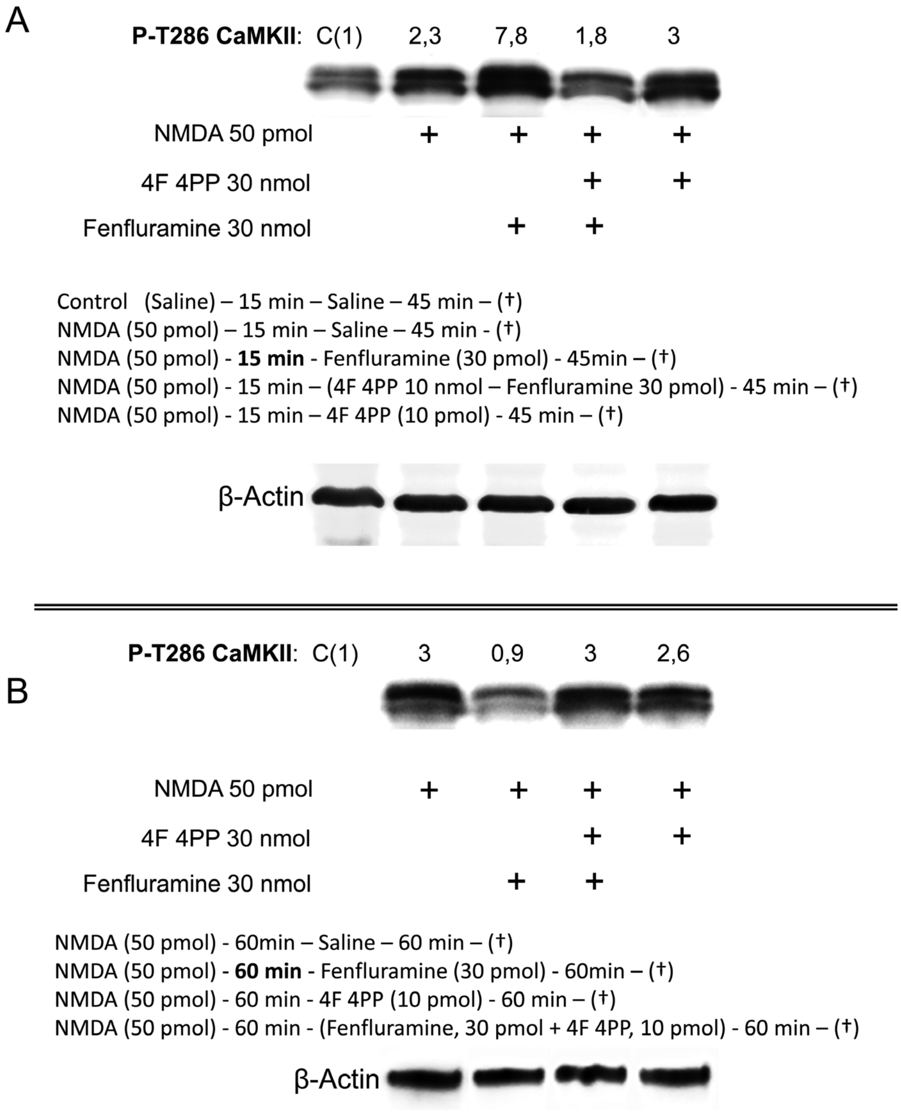

Supplementary Figure 1: Western blot images of fenfluramine effect on NMDA-induced P-T286 CaMKII. Influence of the time interval between these drugs, (**A**) 15 min and (**B**) 60 min). Effect of 4F 4PP, antagonist at 5HT2ARs. Images were visualized by chemiluminescence and recorded using an ImageQuant™ LAS 500. For each blot two areas of capture were typically selected, the target under evaluation and the loading control. The software automatically calculates the optimal exposure time for each of the specified areas to provide the highest possible signal to enable accurate quantification of the sample. Protein immunosignals, and those of actin, were measured using the area of the strongest signal of each studied group of samples (average optical density of the pixels within the object area/mm2; AlphaEase FC software), the grey values of the means were then normalized within the 8 bit/256 grey levels [(256-computed value)/computed value]. Equal loading was verified and adjusted, if necessary, versus actin. The assay was repeated at least twice. The immunosignals are expressed relative to the control (C, value of 1), which received saline instead of the treatments indicated. Details in Methods.

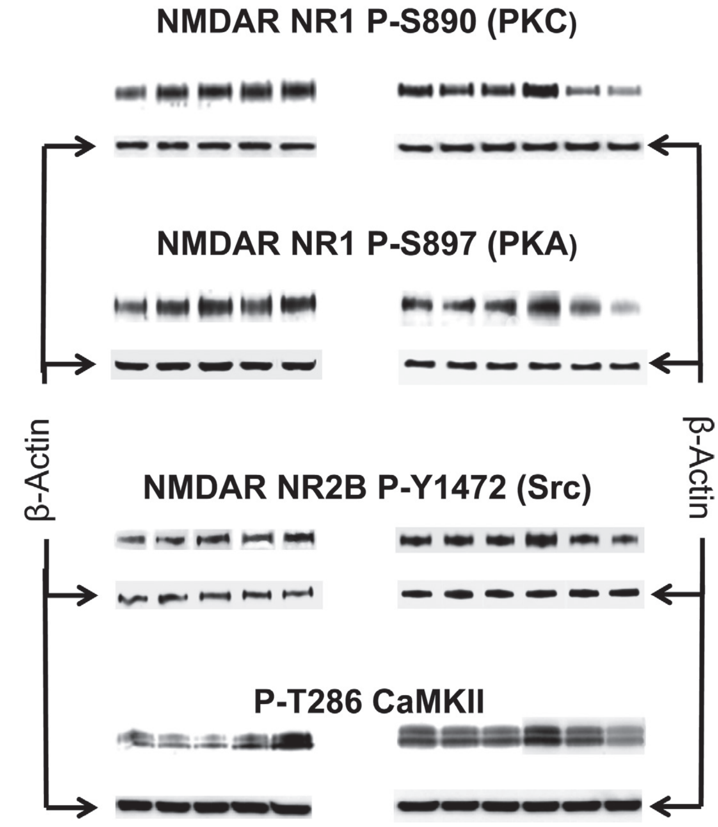

Supplementary Figure 2: Western blot images of Figure 3. Details as in Supplementary Figure 1 and Methods.

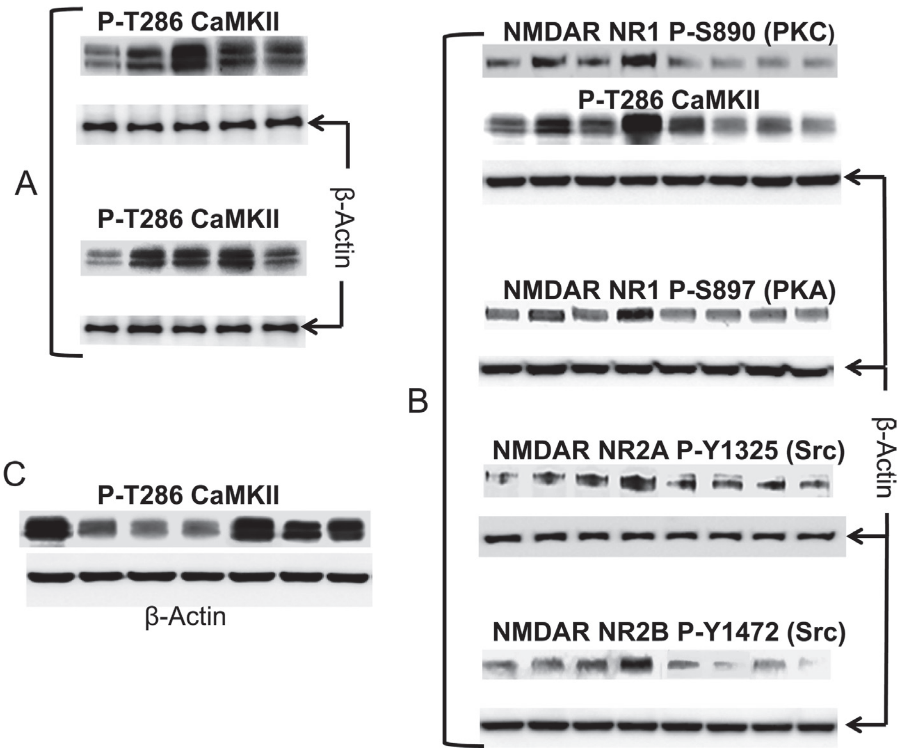

Supplementary Figure 3: Western blot images of Figure 4. Details as in Supplementary Figure 1 and Methods.

Supplementary Video 1: See Supplementary_Video_1

Supplementary Video 2: See Supplementary_Video_2

Supplementary Video 3: See Supplementary_Video_3

Supplementary Video 4: See Supplementary_Video_4

Supplementary Video 5: See Supplementary_Video_5
